# Combination of the glycated hemoglobin levels and prognostic nutritional index as a prognostic marker in patients with acute coronary syndrome and type 2 diabetes mellitus

**DOI:** 10.1186/s12944-023-01992-z

**Published:** 2024-01-11

**Authors:** Shuaifeng Sun, Yue Wang, Shuo Pang, Xiaofan Wu

**Affiliations:** Department of Cardiology, Beijing Anzhen Hospital, Capital Medical University, 2nd Anzhen Road, Chaoyang District, Beijing, 100029 China

**Keywords:** Malnutrition, Prognostic nutritional index, Glycated hemoglobin, Major adverse cardiac or cerebrovascular events, All-cause mortality

## Abstract

**Background:**

Increased susceptibility to malnutrition and inadequate glycemic control are frequently observed in diabetic patients with coronary artery disease. The assessment of malnutrition is performed using the prognosis nutritional index (PNI). The inadequate glycemic control is measured using glycated hemoglobin (HbA1c). However, the combined effect of PNI and HbA1c on the prognosis in diabetic patients with coronary artery disease remains unknown.

**Methods:**

A study was conducted at Beijing Anzhen Hospital and included 2,005 patients diagnosed with type 2 diabetes mellitus (T2DM) accompanied by acute coronary syndrome (ACS) who underwent percutaneous coronary intervention (PCI) from September 2021 to January 2022. Based on the median PNI and HbA1c, we categorized the patients into four groups including high (H)-PNI/low (L)-HbA1c, H-PNI/H-HbA1c, L-PNI/L-HbA1c, and L-PNI/H-HbA1c. Major adverse cardiac and cerebrovascular events (MACCE) were the primary outcome, including all-cause mortality, nonfatal myocardial infarction (MI), and nonfatal strokes.

**Results:**

Throughout a median follow-up of 16.3 months, 73 patients had MACCE, which comprised 36 cases of all-cause mortality. In comparison to the H-PNI, the L-PNI showed an obvious rise in MACCE and all-cause mortality (log-rank *P* = 0.048 and 0.021, respectively) among the H-HbA1c group. Compared to the other groups, the L-PNI/H-HbA1c group exhibited the greatest risk of MACCE (adjusted hazard ratio [aHR]: 2.50, 95% confidence interval [CI] 1.20–5.23, *P* = 0.014) and all-cause mortality (HR: 3.20, 95% CI 1.04–9.82, *P* = 0.042). With the addition of PNI, MACCE and all-cause mortality prediction models performed significantly better in patients with ACS and T2DM after PCI, particularly in those with H-HbA1c levels.

**Conclusions:**

The combination of L-PNI and H-HbA1c is a prognostic marker for MACCE and all-cause mortality in patients diagnosed with ACS and T2DM who underwent PCI. The PNI can serve as an assessment tool of malnutrition in patients with ACS and T2DM accompanied by H-HbA1c who underwent PCI. Therefore, monitoring the long-term change of the PNI deserves attention in clinical practice.

## Background

A minimally invasive method called percutaneous coronary intervention (PCI) is utilized to increase blood flow to the ischemic region by relieving the narrowing or occlusion of the coronary artery. However, despite improvements in treatment approaches and the use of PCI, there is still a high mortality rate associated with acute coronary syndrome (ACS) [1]. Diabetic patients with ACS have higher incidences of recurrent cardiovascular events than those without diabetes mellitus [2]. Identifying modifiable clinical risks is crucial to recognizing high-risk patients and improving their outcomes.

Malnutrition is an irreplaceable prognosticator of poor outcomes in patients with cardiovascular disease (CVD) [3]. The benefit of malnutrition compared to other clinical factors is that it can be modified [4]. However, the lack of an agreed-upon gold-standard method makes it difficult to diagnose malnutrition. Buzby et al. developed the prognostic nutritional index (PNI) for gastrointestinal surgery [5], which was subsequently modified by Onodera et al. [6]. Two previous studies have reported the predictive significance of PNI in patients with diabetes and CVD [7, 8]. Additionally, high glycated hemoglobin (HbA1c) is also related to poor outcomes in patients with diabetes and CVD [9]. Nevertheless, the correlation between the PNI, HbA1c, and outcomes in patients diagnosed with ACS accompanied by T2DM has not been investigated thoroughly after PCI.

Therefore, the purpose of this study is to investigate the combination of PNI and HbA1c on the prognosis in patients with ACS accompanied by T2DM undergoing PCI and the interaction between these two prognostic factors.

## Methods

### Study population

**Fig. 1:**
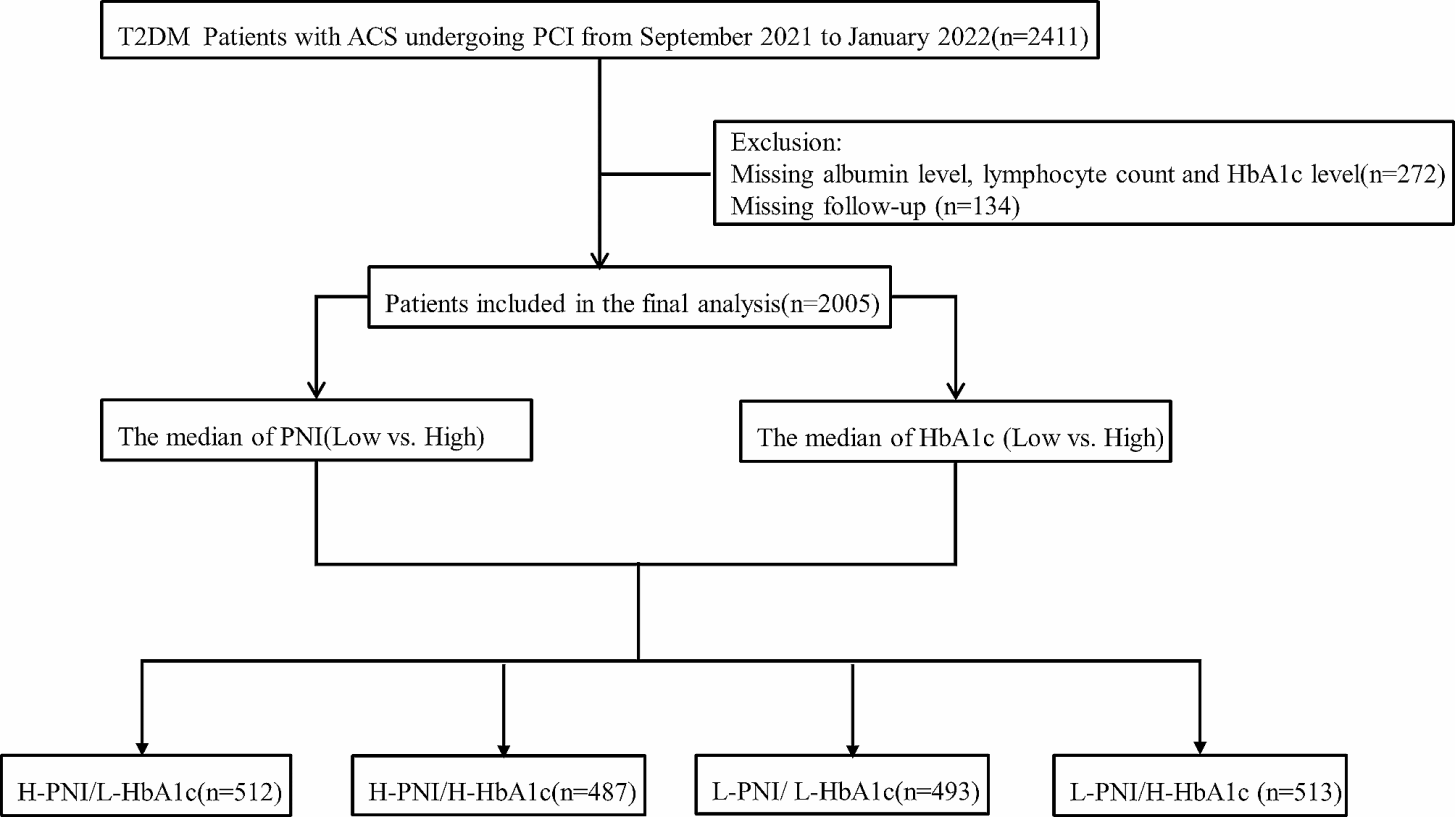
The flowchart of participant selection

A retrospective cohort analysis encompassing 2,411 patients diagnosed with T2DM and ACS who underwent PCI between September 2021 and January 2022 was performed at Beijing Anzhen Hospital. The Electronic Medical Records System of Beijing Anzhen Hospital provided the baseline information at the time of admission. T2DM was defined as the use of oral antidiabetic drugs or insulin or the self-reporting of diabetes [10]. The existing guidelines classified ACS as either unstable angina pectoris (UAP), ST-segment elevation myocardial infarction (STEMI), or non-ST-segment elevation myocardial infarction (NSTEMI) [11]. The PCI was conducted following the standard clinical procedure [12, 13]. Study participants met the following inclusion criteria: (1) at least one clinical phenotype of ACS-UAP, NSTEMI, or STEMI; (2) diagnosis of T2DM; (3) undergoing PCI at Beijing Anzhen Hospital; and (4) complete medical records available, including data on serum albumin (Alb) levels, total lymphocyte counts (TLCs), and HbA1c levels. The exclusion criteria included age under 18 years, PCI failure, chronic liver or renal failure, infectious diseases, taking some drugs that may influence the levels of Alb, and TLCs, and missing clinical data including Alb, TLCs, and HbA1c levels. Patients without measurements of Alb levels, TLCs, or HbA1c levels (n = 272) and those who were missing follow-up data (n = 134) were excluded. Ultimately, the study included 2,005 patients (Fig. 1). All participants in the study provided their informed consent.

### Laboratory data collection

After the patients fasted through the night, their blood samples were collected in the morning and examined in the central laboratory that same day using routine laboratory techniques. An automated blood cell counter was used to count the blood cells. An automatic biochemistry analyzer (Hitachi 7150, Tokyo, Japan) was used to quantify Alb, triglycerides (TG), total cholesterol (TC), low-density lipoprotein cholesterol(LDL-C), high-density lipoprotein cholesterol (HDL-C), uric acid (UA), glucose, creatinine (CR), and the estimated glomerular filtration rate (eGFR). High-performance liquid chromatography was used to measure HbA1c levels (BioRad Variant II TURBO HbA1c analyzer, USA).

### Malnutrition and glycemic control assessment

The PNI was selected as an assessment for malnutrition, and the HbA1c level was used to measure poor glycemic control. The PNI = Alb (g/L) + 5×TLCs (×10^9^/L) [6]. The median PNI and HbA1c levels were 52.9 and 7.0%, respectively. Based on median PNI and HbA1c levels, we divided the patients into four groups: high (H)-PNI/low (L)-HbA1c, H-PNI/H-HbA1c, L-PNI/L-HbA1c, and L-PNI/H-HbA1c.

### Definition of CVD risk factors

Each CVD risk factor and health condition was defined based on well-established criteria. A current smoker was defined as someone who smokes seven cigarettes or more every week for at least 6 months [14]. The 2018 Chinese Heart Failure Guidelines defined heart failure (HF) as New York Heart Association class ≥ III [15]. Chronic kidney disease (CKD) is defined as eGFR of less than 60 mL/min/1.73 m^2^ [16]. The definition of hypertension included self-reported hypertension, and antihypertensive drug usage [17]. To meet the definition of hyperlipidemia, patients had to satisfy at least one of the following criteria: usage of medication to lower lipids or a self-reported record of dyslipidemia [18]. Coronary angiogram characteristics were visually measured and analyzed by a minimum of two experienced cardiologists.

### Endpoints and follow-up

Major adverse cardiac and cerebrovascular events (MACCE) were the primary outcome. All-cause mortality was deemed cardiac if there was no clear noncardiac cause. The identification of MI was conducted in adherence to the Fourth Universal Definition of Myocardial Infarction. The classification of stroke encompassed both ischemic and hemorrhagic stroke. The termination of the follow-up period is contingent upon the earliest occurrence of MACCE, loss of follow-up, or the date of March 10, 2023.

### Statistical analysis

Continuous variables are commonly reported as medians with interquartile ranges or mean values with standard deviations. Categorical variables are commonly expressed as percentages and frequencies. To identify potential disparities among subgroups, statistical tests, such as chi-square and analysis of variance, were employed.

Kaplan–Meier curves were used to examine the likelihood of survival between the groups through log-rank tests. By using Cox proportional hazard models, the hazard ratio (HR) was computed. Variables were chosen based on their established connections with poor prognosis, as determined using a univariate Cox regression analysis. Model 1 remained alone without any modifications. Model 2 was calibrated for age, sex, body mass index (BMI), and current smoking status. Adjusting for the variables obtained from Model 2 and recognized risk factors, including hypertension, hyperlipidemia, prior MI, HF, CKD, and ACS type, was modified in Model 3. Model 4 was altered to include the variables from Model 3, along with TG, TC, and LDL-C levels.

To examine whether adding the PNI to Model 5 (the variables in Model 4 plus HbA1c levels) would improve accuracy in predicting poor outcomes, the concordance index (C-index), net reclassification improvement (NRI), and integrated discrimination improvement (IDI) were collected. In Model 5, we conducted an additional investigation into the relationship between the PNI and poor outcomes using restricted cubic splines.

R software version 4.2.0 (R Foundation for Statistical Computing, Vienna, Austria) was used to perform the statistical analysis. It was determined that the data was significant by using *P* < 0.05.

## Results

### Study population characteristics

Among the 2,005 enrolled patients, 1,717 (85.6%), 182 (9.1%), and 106 (5.3%) had UAP, NSTEMI, and STEMI, respectively. Among all the participants, those with L-PNI/L-HbA1c group were the oldest. More women and participants with lower BMI have been observed in the L-PNI/H-HbA1c group than in the other groups. Patients with worsening renal and cardiac function, as well as comorbidities and prior adverse events, were more common in the groups with L-PNI/H-HbA1c and L-PNI/L-HbA1c. Different groups did not experience significant increases in the severity of coronary lesions (Table 1).

**Table 1:** Baseline characteristics of the study population

Variable	H-PNI/L-HbA1c (n = 512)	H-PNI/H-HbA1c (n = 487)	L-PNI/ L-HbA1c (n = 493)	L-PNI/H-HbA1c (n = 513)	*P*
Age (years)	58.5 ± 9.8	58.7 ± 10.3	64.0 ± 8.7	61.9 ± 9.0	< 0.001
Women, n (%)	125(24.4)	144(29.6)	133(27.0)	177(34.5)	0.03
BMI (kg/m^2^)	26.5 ± 3.3	26.4 ± 3.3	25.8 ± 3.1	25.9 ± 3.2	< 0.001
**Comorbidities, n (%)**					
Hypertension	383(74.8)	332(68.2)	374(75.9)	371(72.3)	0.034
Hyperlipidemia	427(83.4)	430(88.3)	387(78.5)	439(85.6)	< 0.001
Current smoker	163(31.8)	151(31)	119(24.1)	131(25.5)	0.011
Prior MI	69(13.5)	72(14.8)	101(20.5)	87(17)	0.017
Prior PCI	176(34.4)	160(32.9)	192(38.9)	187(36.5)	0.212
Prior stroke	32(6.3)	42(8.6)	52(10.5)	52(10.1)	0.068
HF	14(2.7)	20(4.1)	30(6.1)	34(6.6)	0.014
CKD	14(2.7)	22(4.5)	51(10.3)	34(6.6)	< 0.001
PAD	14(2.7)	18(3.7)	23(4.7)	18(3.5)	0.44
**ACS type, n (%)**					
STEMI	19(3.7)	19(3.9)	18(3.7)	50(9.7)	< 0.001
NSTEMI	33(6.4)	34(7.0)	47(9.5)	68(13.3)	< 0.001
UAP	460(89.9)	434(89.1)	428(86.8)	397(77.0)	< 0.001
**Procedure characteristics**					
Multivessel disease, n (%)	381(74.4)	363(74.5)	381(77.3)	397(77.4)	0.523
Left main lesion, n (%)	29(5.7)	37(7.6)	45(9.1)	31(6)	0.125
Bifurcation lesion, n (%)	67(13.1)	47(9.7)	45(9.1)	67(13.1)	0.18
Chronic total occlusion, n (%)	57(11.1)	64(13.1)	72(14.6)	63(12.3)	0.409
Stent number	1.6 ± 1.0	1.6 ± 0.9	1.6 ± 0.9	1.6 ± 0.9	0.623
Total stent length (mm)	30(20,48.5)	32(20,51)	32(20,51)	30(20,50)	0.761
**Basic laboratory data**					
TLCs (×10/^9^L)	2.1 ± 1.1	2.2 ± 0.6	1.4 ± 0.4	1.5 ± 0.4	< 0.001
HGB (g/L)	145.3 ± 14.9	145.6 ± 15.3	135.1 ± 17.2	137.4 ± 16.7	< 0.001
Alb (g/L)	47.0 ± 2.9	46.4 ± 2.8	42.4 ± 3.0	41.8 ± 3.2	< 0.001
TG (mmol/L)	1.6(1.2,2.1)	1.7(1.3,2.4)	1.4(1.1,1.9)	1.5(1.1,2.2)	< 0.001
TC (mmol/L)	4.0 ± 1	4.3 ± 1.1	3.9 ± 0.9	4.2 ± 1.1	< 0.001
LDL-C (mmol/L)	2.1 ± 0.8	2.3 ± 0.9	2.1 ± 0.8	2.3 ± 0.9	< 0.001
HDL-C (mmol/L)	1.1 ± 0.3	1.0 ± 0.2	1.0 ± 0.3	1.0 ± 0.3	0.051
Glucose (mmol/L)	6.7(5.8,7.8)	8.6(6.9,11.2)	6.7(5.7,7.9)	9.0(6.9,12.3)	< 0.001
HbA1c (%)	6.4 ± 0.5	8.3 ± 1.0	6.4 ± 0.5	8.4 ± 1.1	< 0.001
CR (µmol/L)	75.9(66.0,88.1)	76.3(67.3,87.3)	77.8(66.6,90.8)	78.0(66.0,91.6)	0.03
eGFR (ml/min per 1.73 m^2^)	92.6(66.0,91.6)	92.2(76,102.0)	87.3(70.8,95.8)	86.6(70.9,98.2)	< 0.001
UA (µmol/L)	330.5 ± 83.4	330.5 ± 96.8	332.3 ± 97.4	319 ± 89.7	0.002
LVEF (%)	61.7 ± 6.8	60.9 ± 7.6	60.1 ± 8	59.1 ± 9	< 0.001
PNI	56.4(54.7,59.0)	56.7(54.9,59)	56.4(48.1,51.8)	50.1(47.6,51.6)	< 0.001
**Pharmacotherapy, n (%)**					
Aspirin	503(98.2)	474(97.3)	484(98.2)	503(98.1)	0.729
Clopidogrel	318(62.1)	301(61.8)	335(68)	341(66.5)	0.1
Ticagrelor	197(38.5)	192(39.4)	161(32.7)	179(34.9)	0.095
Statin	511(99.8)	485(99.6)	491(99.6)	511(99.6)	0.925
ACEI/ARB	232(45.3)	186(38.2)	200(40.6)	218(42.5)	0.132
β-Blocker	357(69.7)	349(71.7)	317(64.3)	340(66.3)	0.057
CCB	196(38.3)	170(34.9)	192(38.9)	176(34.3)	0.314

*Abbreviations*: BMI, body mass index; MI, myocardial infarction; PCI, percutaneous coronary intervention; HF, Heart failure; CKD, Chronic kidney disease; PAD, Peripheral arterial disease; ACS, acute coronary syndrome; STEMI, ST-segment elevation myocardial infarction; NSTEMI, non–ST-segment elevation myocardial; UAP, unstable angina; infarction; TLCs, total lymphocyte count; HGB, hemoglobin; Alb, serum albumin; TG, triglycerides; TC, total cholesterol; LDL-C, low-density lipoprotein cholesterol; HDL-C, high-density lipoprotein cholesterol; HbA1c, glycated hemoglobin; CR, creatinine; eGFR, estimated glomerular filtration rate; UA, uric acid; LVEF, left ventricular ejection fraction; PNI, prognostic nutritional index; ACEI, angiotensin-converting enzyme inhibitor; ARB, angiotensin receptor blocker; CCB, calcium-channel antagonist

### Effect of the PNI and HbA1c levels on clinical endpoint

**Fig. 2:**
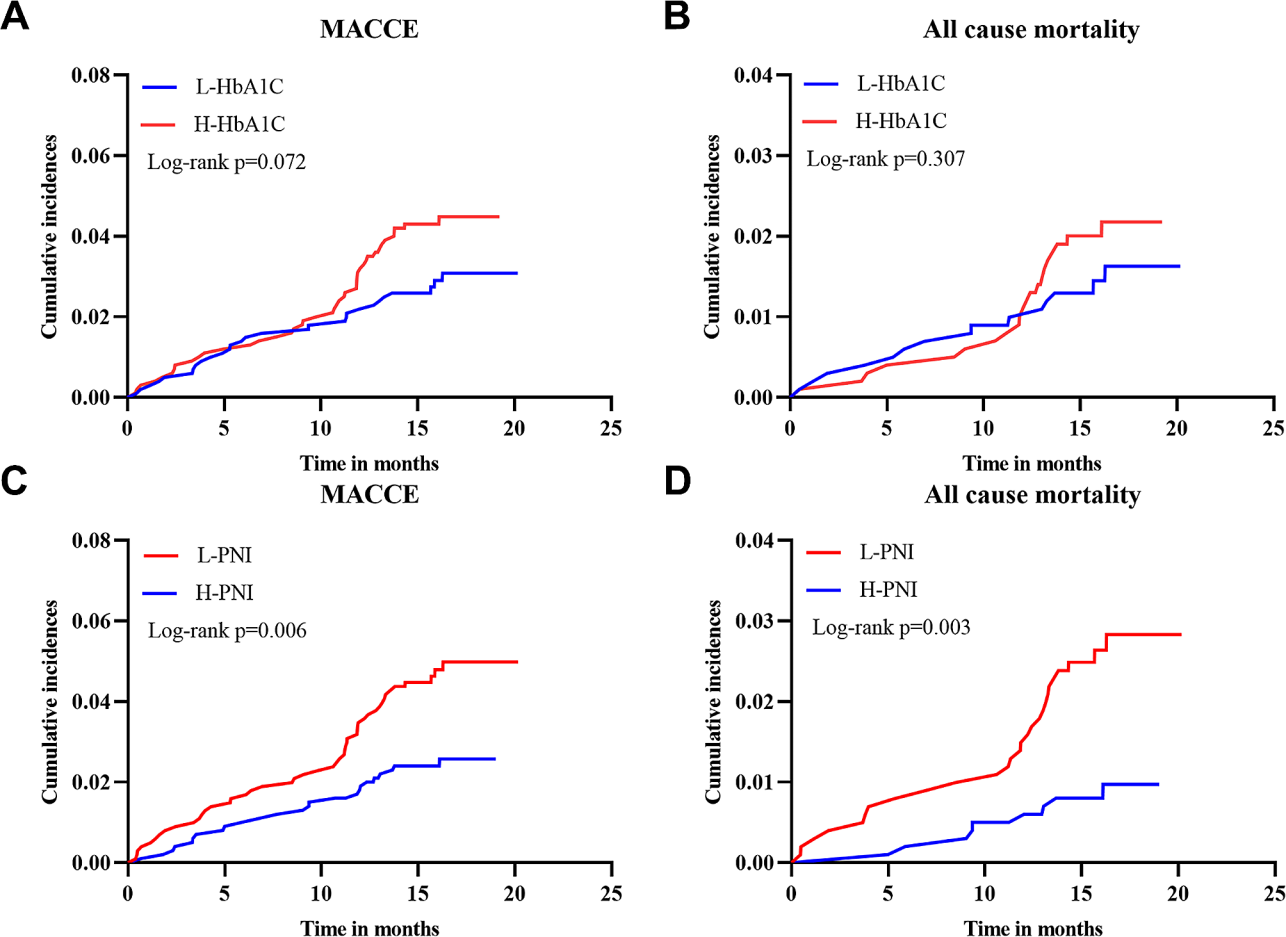
Kaplan–Meier analysis for MACCE and all-cause mortality categorized based on HbA1c and PNI. (**A**) Kaplan–Meier curves of MACCE categorized based on HbA1c levels; (**B**) Kaplan–Meier curves of all-cause mortality categorized based on HbA1c levels; (**C**) Kaplan–Meier curves of MACCE categorized according to PNI; (**D**) Kaplan–Meier curves of all-cause mortality categorized based on PNI. PNI, prognostic nutritional index; HbA1c, glycated hemoglobin; MACCE, major adverse cardiac and cerebrovascular events

**Fig. 3:**
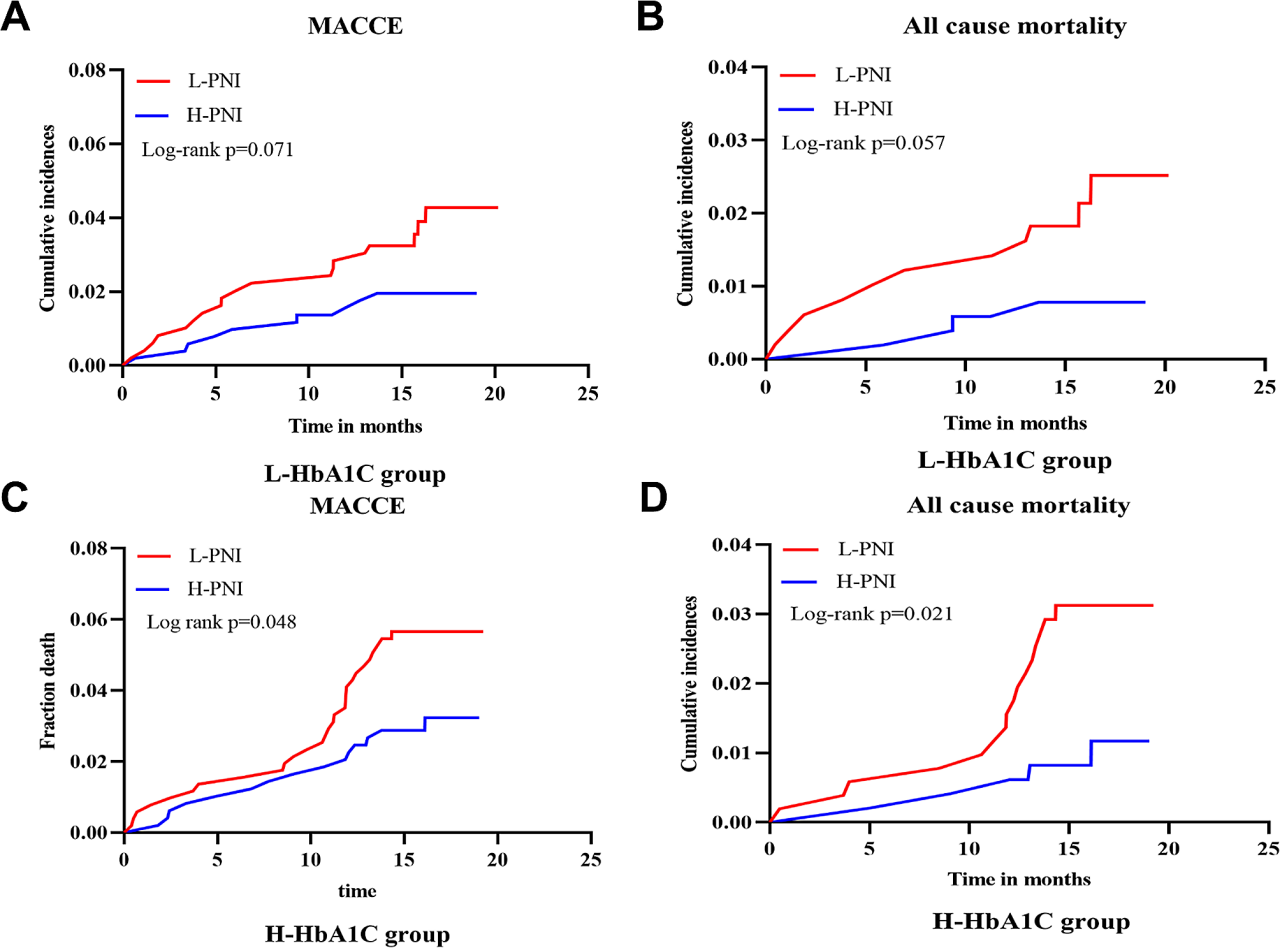
Kaplan–Meier analysis for MACCE and all-cause mortality categorized based on PNI in different subgroups of HbA1c as follows: the L-HbA1c group (**A**, **B**), and the H-HbA1c group (**C**, **D**). PNI, prognostic nutritional index; HbA1c, glycated hemoglobin; MACCE, major adverse cardiac and cerebrovascular events

**Fig. 4:**
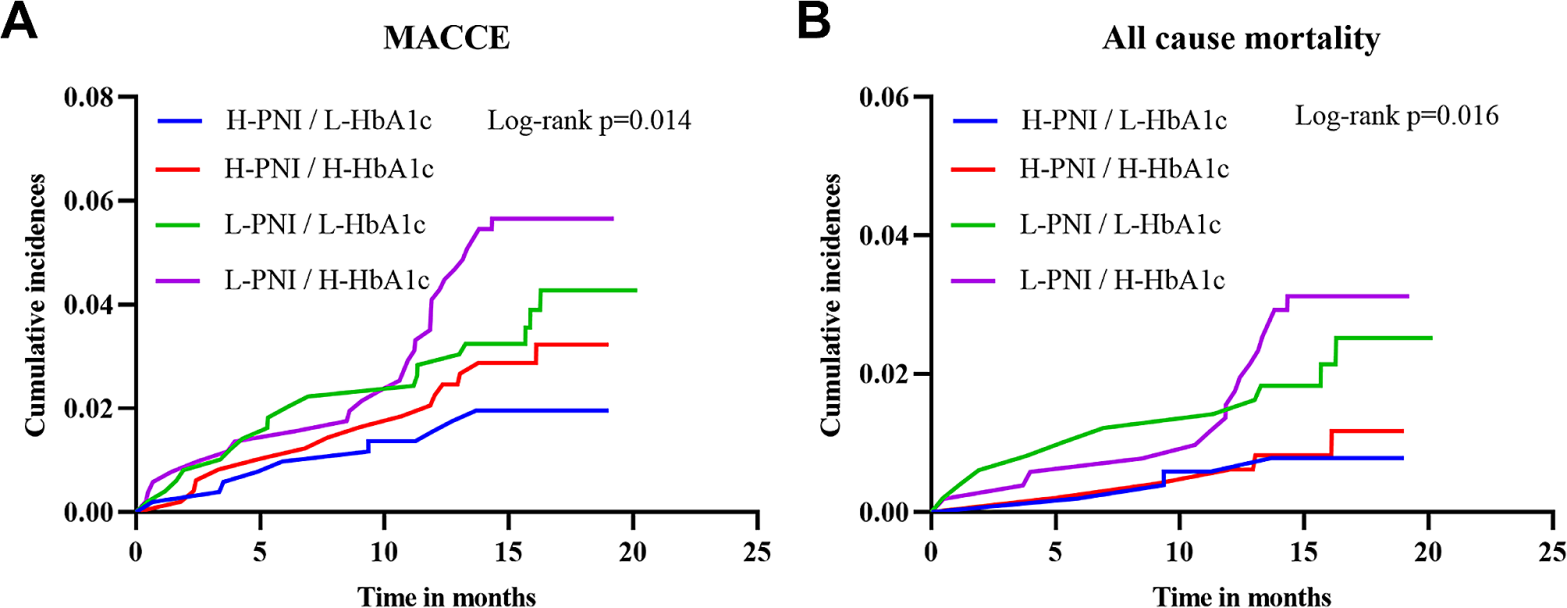
Kaplan–Meier analysis for MACCE (**A**) and all-cause mortality (**B**) categorized based on PNI and HbA1c. PNI, prognostic nutritional index; HbA1c, glycated hemoglobin; MACCE, major adverse cardiac and cerebrovascular events

Throughout a median follow-up of 16.3 months, 73 patients had MACCE, including 36, 17, and 20 cases of all-cause mortality, nonfatal MI, and nonfatal stroke, respectively. Based on Kaplan–Meier curves, L-PNI had a significantly higher association with MACCE and all-cause mortality than H-PNI in the overall study population (log-rank *P* = 0.006 and 0.003, respectively) (Fig. 2C, D), while no significant statistical association was found between H-HbA1c and MACCE or all-cause mortality (Fig. 2A, B). L-PNI demonstrated better performance in predicting MACCE and all-cause mortality than the H-PNI (log-rank *P* = 0.048 and 0.021, respectively) in the H-HbA1c group (Fig. 3C, D). In contrast, no statistical significance was found for the L-HbA1c group (log-rank *P* > 0.05 for both) (Fig. 3A, B). Additionally, Kaplan–Meier curves comparing all four groups revealed that the L-PNI/H-HbA1c group had the highest MACCE (log-rank *P* = 0.014) and all-cause mortality (log-rank *P* = 0.016), compared to the other groups (Fig. 4A, B).

After adjusting for the variables of Model 4, the modified Cox proportional hazard models indicated that patients with L-PNI/H-HbA1c showed the greatest risk of all-cause mortality (adjusted hazard ratio [aHR] 3.20, 95% confidence interval [CI] 1.04–9.82, *P* = 0.042) and MACCE (aHR 2.50, 95%CI 1.20–5.23, *P* = 0.014) and in comparison to the remaining groups. The other two groups were not related to a significantly increased risk (Table 2).

**Table 2:** Cox proportional hazard models for MACCE and all-cause mortality

Groups	Model 1		Model 2		Model 3		Model 4	
MACCE	HR (95%CI)	*P*	HR (95%CI)	*P*	HR (95%CI)	*P*	HR (95%CI)	*P*
H-PNI/L-HbA1c	1.00 (reference)	/	1.00 (reference)	/	1.00 (reference)	/	1.00 (reference)	/
H-PNI/H-HbA1c	1.59(0.71–3.53)	0.259	1.59(0.71–3.53)	0.258	1.52(0.68–3.39)	0.307	1.51(0.68–3.39)	0.313
L-PNI/ L-HbA1c	2.00(0.93–4.29)	0.077	1.82(0.84–3.95)	0.128	1.56(0.71–3.39)	0.265	1.60(0.73–3.48)	0.239
L-PNI/H-HbA1c	2.94(1.44–6.04)	0.003	2.80(1.36–5.77)	0.005	2.47(1.18–5.14)	0.016	2.50(1.20–5.23)	0.014
**All-cause mortality**								
H-PNI/L-HbA1c	1.00 (reference)	/	1.00 (reference)	/	1.00 (reference)	/	1.00 (reference)	/
H-PNI/H-HbA1c	1.32(0.35–4.90)	0.682	1.31(0.35–4.88)	0.687	1.26(0.34–4.71)	0.731	1.29(0.34–4.84)	0.708
L-PNI/ L-HbA1c	2.89(0.92–9.07)	0.069	2.48(0.78–7.86)	0.123	2.03(0.64–6.48)	0.231	2.06(0.64–6.58)	0.223
L-PNI/H-HbA1c	4.05(1.35–12.10)	0.012	3.67(1.22–11.05)	0.02	3.14(1.03–9.60)	0.044	3.20(1.04–9.82)	0.042

Data are presented as HR (hazard ratio), 95% CI (confidence intervals), and *P*-value

Model 1: unadjusted

Model 2: adjusted for Age, Sex, BMI, and Current smoker

Model 3: adjusted for Age, Sex, BMI, Current smoker, Hypertension, Hyperlipidemia, Prior MI, HF, CKD, ACS type

Model 4: adjusted for Age, Sex, BMI, Current smoker, Hypertension, Hyperlipidemia, Prior MI, HF, CKD, ACS type, TG, TC, LDL-C

### Model discrimination

**Fig. 5:**
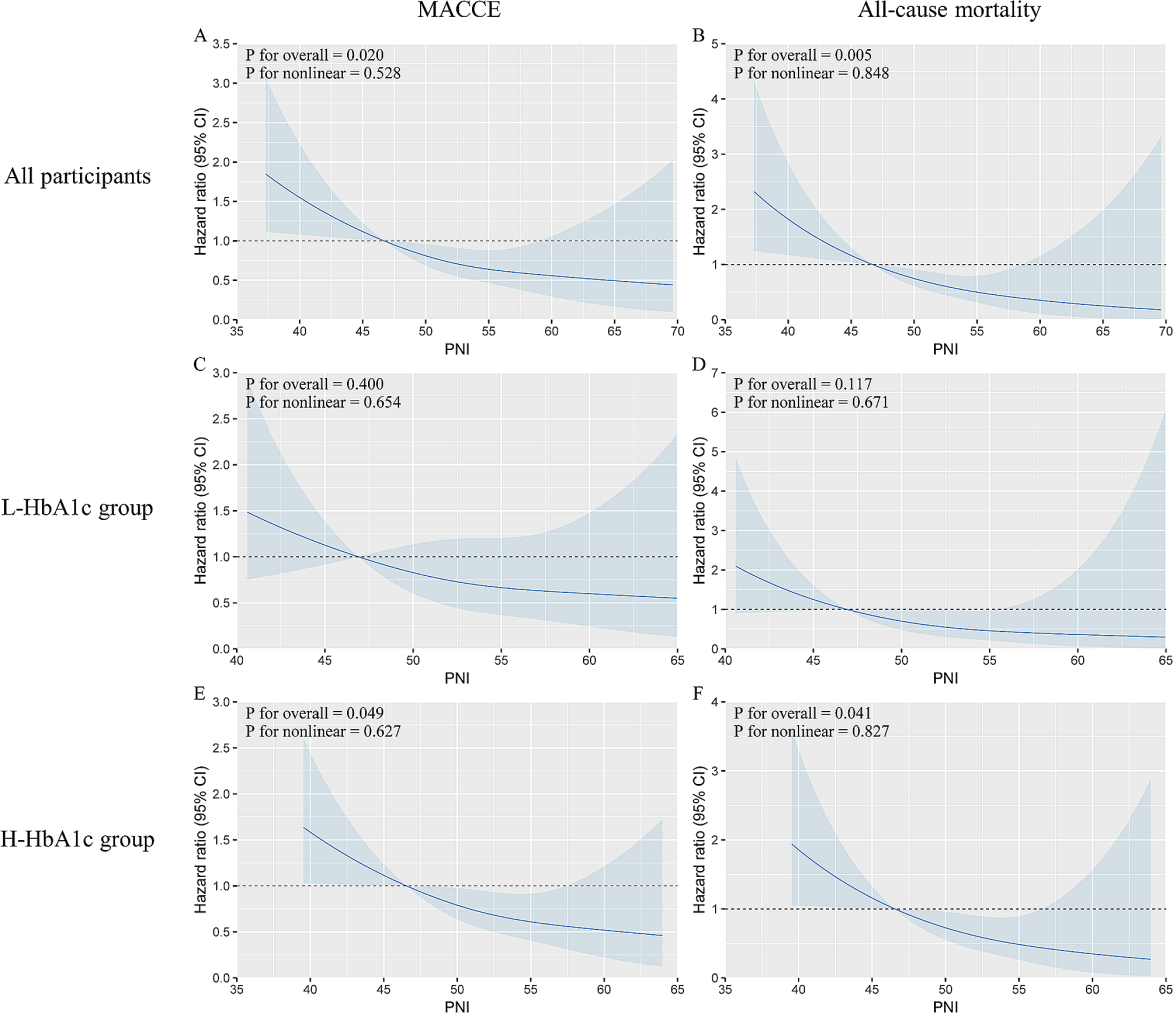
Restricted spline curves for the associations between PNI, MACCE, and all-cause mortality stratified by all participants (**A**, **B**), L-HbA1c group (**C**, **D**), and H-HbA1c group (**E**, **F**). PNI, prognostic nutritional index; HbA1c, glycated hemoglobin; MACCE, major adverse cardiac and cerebrovascular events

Table 3 shows that incorporating PNI into Model 5 improved the prediction of MACCE (increase in C-index from 0.664 to 0.681, *P* = 0.24; NRI 0.023, *P* = 0.023; IDI 0.005, *P* = 0.023) and all-cause mortality (increase in C-index from 0.727 to 0.763, *P* = 0.176; NRI 0.527, *P* < 0.001; IDI 0.007, *P* = 0.014;) in the overall study population. In patients with L-HbA1c, however, this improvement was not statistically significant (Table 3). MACCE, all-cause mortality, and continuous PNI showed a significant linear connection in patients with H-HbA1c levels (Fig. 5E, F); however, patients with L-HbA1c showed no significant association with there parameters (Fig. 5C, D).

**Table 3:** Evaluation of predictive models for MACCE and all-cause mortality

	C-index (95%CI)	*P*	NRI (95%CI)	*P*	IDI (95%CI)	*P*
**All participants**						
*MACCE*						
Established factors	0.664(0.598–0.731)	Reference	/	Reference	/	Reference
Established factors + PNI	0.681(0.614–0.748)	0.24	0.266(0.035–0.497)	0.023	0.005(0–0.010)	0.023
*All-cause mortality*						
Established factors	0.727 0.638–0.817)	Reference	/	Reference	/	Reference
Established factors + PNI	0.763(0.676–0.849)	0.176	0.527(0.216–0.838)	< 0.001	0.007(0.001–0.013)	0.014
**L-HbA1c group**						
*MACCE*						
Established factors	0.726(0.633–0.819)	Reference	/	Reference	/	Reference
Established factors + PNI	0.724(0.626–0.822)	0.917	0.183(−0.184 to 0.550)	0.327	0.003(−0.001 to 0.008)	0.133
*All-cause mortality*						
Established factors	0.725(0.591–0.858)	Reference	/	Reference	/	Reference
Established factors + PNI	0.743(0.605–0.881)	0.72	0.404(−0.095 to 0.903)	0.112	0.008(−0.002 to 0.018)	0.124
**H-HbA1c group**						
*MACCE*						
Established factors	0.686(0.604–0.768)	Reference	/	Reference	/	Reference
Established factors + PNI	0.694(0.610–0.778)	0.665	0.331(0.037–0.626)	0.027	0.009(0–0.019)	0.034
*All-cause mortality*						
Established factors	0.765(0.677–0.854 )	Reference	/	Reference	/	Reference
Established factors + PNI	0.796(0.714–0.879)	0.257	0.534(0.126–0.942)	0.01	0.011(0–0.023)	0.053

Established risk factors included Age, Sex, BMI, Current smoker, Hypertension, Hyperlipidemia, Prior MI, HF, CKD, ACS type, TG, TC, LDL-C, and HbA1c. 95% CI, 95% confidence interval; IDI, integrated discrimination improvement; NRI, net reclassification improvement; PNI, prognostic nutritional index

## Discussion

This is the initial investigation to examine the correlation between PNI and HbA1c levels, as well as the poor outcomes in patients diagnosed with T2DM and ACS who underwent PCI and the interaction between the two prognostic factors. The main findings can be summarized as follows: (1) L-PNI had a significant effect on MACCE and all-cause mortality in the overall patients; (2) For MACCE and all-cause mortality, H-HbA1c exacerbated the negative impact of L-PNI; (3) patients in the L-PNI/H-HbA1c group encountered the highest rates of MACCE and all-cause mortality; and (4) incorporating PNI significantly improved model performance for predicting MACCE and all-cause mortality in all patients, particularly in individuals with H-HbA1c levels. The results highlight the importance of the PNI and its interaction with HbA1c levels in patients with ACS accompanied by T2DM who underwent PCI.

Previous research has demonstrated that there is an important association between malnutrition and poor outcomes in patients with ACS or T2DM [19, 20]. Various nutritional screening tools have been proposed, apart from the PNI, such as the geriatric nutritional risk index (GNRI), the subjective global assessment (SGA), the controlling nutritional status (CONUT) score, TG-TC-body weight index (TCBI), and mini-nutritional assessment (MNA), each of which has advantages and disadvantages. Due to the subjectivity of the parameters used, the clinical experience of the evaluators may influence the results of SGA or MNA [21]. With statins being widely used in patients with CVD, the measurement of the CONUT score and TCBI may be affected [22, 23]. Since the GNRI uses body weight for calculation, patients with normal or high BMI can be underestimated as malnourished [24]. Additionally, the PNI was found to be more accurate than GNRI, TCBI, and COUNT scores in predicting mortality in the general population [25]. Compared to the other assessments, the PNI is an easy and efficient screening tool that uses widely available markers of malnutrition. Therefore, the PNI was chosen to evaluate malnutrition of patients with ACS complicated with T2DM who underwent PCI.

Consistent with previous findings [19, 26], poor nutritional status, defined in the study as L-PNI, was related to poor prognosis in patients with ACS. The included patients who were L-PNI/H-HbA1c had the highest risk of MACCE and all-cause mortality. H-HbA1c aggravated the adverse effects of L-PNI. A significant negative nitrogen balance is frequently linked to H-HbA1c levels in patients with diabetes. The unevenness is partially caused by increased protein degradation and elimination, along with reduced protein production. Therefore, an increased risk of malnutrition exists [27], and it further worsens insulin resistance, negatively impacting the overall health of the patient. Contrary to patients in the H-PNI/H-HbA1c, patients in the L-PNI/H-HbA1c faced an increased likelihood of MACCE and all-cause mortality, consistent with prior research suggesting that malnutrition is more widespread than other long-term comorbidities, underscoring the importance of PNI [7]. HbA1c levels are typically managed actively, whereas subclinical PNI is typically not managed. This may explain the differential impact of these two factors on poor prognosis. H-HbA1c enhanced the detrimental impact of L-PNI on the prognosis in patients with diabetes, possibly because of their different underlying mechanisms. Patients with DM with H-HbA1c have a greater chance of experiencing each distinct disease entity when inflammation and malnutrition are combined [22]. It is also possible that malnutrition aggravates systemic inflammation in patients with diabetes, leading to poor outcomes [28, 29]. Overall, inflammation and malnutrition are significant predictors of poor prognosis after PCI in patients with ACS complicated with T2DM.

Therefore, incorporating malnutrition screening into daily clinical practice is essential for patients with ACS. By implementing malnutrition screening, patients who are at an elevated risk are identified and provided with targeted secondary prevention interventions [30]. Malnutrition can be prevented and treated using various multidisciplinary strategies, including oral nutritional supplements, fortification or enrichment of foods or fluids, and dietary counseling [31]. Along with other intensive lifestyle changes, dietary interventions have been associated with significant reductions in coronary atherosclerosis [32]. Furthermore, preventing malnutrition may be even more crucial in patients with ACS and T2DM to avoid a deterioration in their nutritional status and general health.

### Study strengths and limitations

This research has several strengths. First, an adequate sample size was used in the study, which was performed at Beijing Anzhen Hospital, the biggest cardiovascular center in China. Second, the relationship between PNI and HbA1c on the prognosis of patients with ACS accompanied by T2DM undergoing PCI was explored for the first time. Third, the present investigation revealed that patients’ nutritional state should be taken seriously, especially if they have elevated H-HbA1c levels. Nevertheless, there were certain limitations associated with this research. First, the fact that this single-center study which is restricted to the Chinese population limits the applicability of these findings to other populations. Second, PNI changes over time may impact poor outcomes; however, this is unclear. Third, the lack of data pertaining to education, income, and dietary patterns limits our understanding of the fundamental determinants that contribute to the prevalence of malnutrition. Fourth, the information on CRP values was not available in the current database. Finally, no comparison was made regarding the predictive significance of various nutritional assessment instruments.

## Conclusions

The correlation between L-PNI and H-HbA1c is a vital indicator for MACCE and all-cause mortality in patients diagnosed with ACS and T2DM following PCI. The PNI can serve as an evaluation for the risk of malnutrition, particularly in those with H-HbA1c levels. Furthermore, monitoring malnutrition through the PNI could better identify patients susceptible to cardiovascular events.

## Data Availability

The data will not be shared, because the identified participant information is included in the data.

## Abbreviations

PNI: prognostic nutritional index
HbA1c: glycated hemoglobin
MACCE: major adverse cardiac and cerebrovascular events
T2DM: type 2 diabetes mellitus
PCI: percutaneous coronary intervention
ACS: acute coronary syndrome
CVD: cardiovascular disease
UAP: unstable angina pectoris
NSTEMI: non-ST-segment elevation myocardial infarction
STEMI: ST-segment elevation myocardial infarction
Alb: albumin
TLCs: total lymphocyte counts
TG: triglycerides
TC: total cholesterol
LDL-C: low-density lipoprotein cholesterol
HDL-C: high-density lipoprotein cholesterol
HGB: hemoglobin
eGFR: estimated glomerular filtration rate
HF: heart failure
CKD: chronic kidney disease
MI: myocardial infarction
BMI: body mass index
NRI: net reclassification improvement
IDI: integrated discrimination improvement
CI: confidence interval
HR: hazard ratio
SGA: subjective global assessment
MNA: mini-nutritional assessment
CONUT: controlling nutritional status
TCBI: TG-TC-body weight index
GNRI: geriatric nutritional risk index
